# ORION: a web server for protein fold recognition and structure prediction using evolutionary hybrid profiles

**DOI:** 10.1038/srep28268

**Published:** 2016-06-20

**Authors:** Yassine Ghouzam, Guillaume Postic, Pierre-Edouard Guerin, Alexandre G. de Brevern, Jean-Christophe Gelly

**Affiliations:** 1 INSERM, U 1134, DSIMB, F-75739 Paris, France; 2 Univ. Paris Diderot, Sorbonne Paris Cité, UMR_S 1134, F-75739 Paris, France; 3 Institut National de la Transfusion Sanguine (INTS), F-75739 Paris, France; 4 Laboratoire d’Excellence GR-Ex, F-75739 Paris, France

## Abstract

Protein structure prediction based on comparative modeling is the most efficient way to produce structural models when it can be performed. ORION is a dedicated webserver based on a new strategy that performs this task. The identification by ORION of suitable templates is performed using an original profile-profile approach that combines sequence and structure evolution information. Structure evolution information is encoded into profiles using structural features, such as solvent accessibility and local conformation —with Protein Blocks—, which give an accurate description of the local protein structure. ORION has recently been improved, increasing by 5% the quality of its results. The ORION web server accepts a single protein sequence as input and searches homologous protein structures within minutes. Various databases such as PDB, SCOP and HOMSTRAD can be mined to find an appropriate structural template. For the modeling step, a protein 3D structure can be directly obtained from the selected template by MODELLER and displayed with global and local quality model estimation measures. The sequence and the predicted structure of 4 examples from the CAMEO server and a recent CASP11 target from the ‘Hard’ category (T0818-D1) are shown as pertinent examples. Our web server is accessible at http://www.dsimb.inserm.fr/ORION/.

Proteins are major biological macromolecules involved in many critical processes. The three dimensional (3D) structure of a protein determines its function, which makes obtaining of protein 3D structures essential for functional and evolutionary studies. Despite the efficiency of experimental methods (X-ray crystallography, NMR spectroscopy, and cryo-EM) to determine the 3D structure of proteins, these techniques are still costly and time-consuming. Moreover, the number of resolved protein structures is growing at a slower rate than the number of protein sequences in databanks (from 2008 to 2016: +1000% protein sequences and +100% protein structures)^1^,^2^. In this context, *in silico* approaches of protein structure modeling and prediction are a solution to access 3D information directly from sequence. Template-based modeling is currently the main method for protein structure prediction^3^,^4^. Protein homology/analogy detection between a query and a template protein having a resolved structure is a crucial part in this strategy^5^. Nonetheless, an important part of distant relationships are not detectable by classical sequence search methods and more sensitive approaches must be employed.

Initially, remote homology detection approaches relied on profile-to-sequence comparison^6^. A profile is a position-specific scoring matrix (PSSM) obtained from multiple sequence alignment of homologous proteins. Thus, it contains evolutionary information specific to a protein family encoded by the levels of residue conservation at each sequence position. PSI-BLAST^7^ was the first method to use the profile-to-sequence algorithm proposed by Henikoff and Henikoff^8^. Profile-to-sequence comparisons have led to improvement of the remote homology detection but other improvements were made using profiles based on hidden Markov models (HMMs profiles)^9^,^10^,^11^, which allow a probabilistic interpretation of inserts and deletions along the alignment. A new generation of fold recognition methods has been introduced with the Fold and Function Assignment System method (FFAS)^12^, which was based on profile–profile comparisons. Theses approaches take the full advantages of the transitivity of sequence homology by using profiles for both target and template and, therefore, become more sensitive than profile-to-sequence alignments^13^,^14^,^15^.

Finally, the pairwise profile HMM comparison performed by the HHsearch algorithm^16^ has further increased the sensitivity and specificity detection of remote homologous proteins. Compared with sequence-to-sequence and profile-to-sequence approaches, profile and profile HMMs pairwise comparisons improved comparative modeling through enhanced template identification and alignment quality^17^,^18^. It has been shown that the accuracy of these methods could be improved with the incorporation of accurate local structural features since proteins might have structural similarities even when no evolutionary relationship of their sequences can be detected^12^,^18^,^19^. Several methods combining discrete structural features, such as solvent accessibility and secondary structure, with amino acid sequence information have been proposed, e.g. 3D-PSSM^20^ or FUGUE^21^. Since structure is three to ten times more conserved than sequence throughout evolution^19^, structural information would be more conserved and richer in evolutionary information than sequence information. Therefore, combining sequence and structure information into a hybrid profile is a better approach for the detection of distant homology relationships^22^.

ORION is a fold recognition method based on the pairwise comparison of profiles combining sequence and structural information recently developed in our group^22^. It relies on a better description of the local protein structure to boost distantly protein detection. These descriptors called Protein Blocks (PB) encode a structural alphabet defined by 16 local structural patterns that accurately describe local protein structures^23^. PB is currently the most widely used structural alphabet^24^. Thanks to PB structural descriptor and hybrid profile-profile comparisons, ORION outperforms, in terms of template detection sensitivity at fold level, profile-sequence methods like PSI-BLAST by 16% more and profile-profile methods like HHsearch by 5% more^22^.

Recently, we have improved our ORION method by adding solvent accessibility as a new structural feature, which improves template detection by more than 5% compared to the initial version. We present here the ORION web server, freely usable for scientific and academic community, along with our new and improved approach.

## Methods

### ORION algorithm

As with all profile-profile methods, ORION algorithm is divided into three main steps: (i) preparation of the multiple sequence alignment (MSA) of query -potential- homologs, (ii) generation of query profile and (iii) alignment of the query profile to templates profiles from a databank.

In the first step, MSA is obtained by three iterations of PSI-BLAST on the non-redundant databank Uniref90^25^ with an E-value threshold of 10^−4^. Then in the next step, the query amino acid profile (AA profile) is derived from the MSA. It contains the probabilities of each of the 20 amino acids plus an additional probability that describes the gap frequency at this position. Two structural profiles are predicted from this MSA: the Protein Blocks profile (PB profile) and the solvent accessibility profile (SA profile). The PB profile is predicted using a similar approach to LOCUSTRA^26^, namely a two layer support vector network with the AA profile. This PB profile contains the probabilities of the 16 PB letters at each position. The SA profile is obtained from the solvent accessibility predicted for each residue by PROF software^27^ (see recent improvements section).

In the last step, the AA, PB and SA query profiles are concatenated to search the selected databank of AA/PB/SA template profiles. These template profiles have been pre-calculated and contain information of PB and solvent accessibility features computed from the protein 3D coordinates, with a homemade Python script for PB assignment and NACCESS^28^,^29^ for solvent accessibility. The databank search is then performed using ORION software^22^.

### Recent improvements

We have improved the initial version of ORION with three main novelties. First is the inclusion of position specific gap penalties in the method. Since conserved residues in the alignment should accept fewer gaps than those that are not conserved, we have added a gap position to profiles that describes gap probability at each position for a more accurate alignment.

Secondly, we have appended a correlation score to the ORION scoring system. Indeed, Pei *et al*. have shown that alignments of homologous sequences tend to have clusters of conserved columns along the sequence^30^. When two homolog profiles are aligned, conserved columns should also occur in clusters along the alignment. Thus, we integrated a correlation score to ORION scoring system in the same way as in HHsearch^16^.

The correlation score (*S*_*corr*_) is described in equations (1, 2) with *S*_*l*_ corresponding to the score of the *l*th position of the alignment. Suppose *L* is the length of the alignment between the query and template profile. *S*_*corr*_ is the correlation score *S*_*l*_ over a sliding window of length *d*.


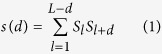



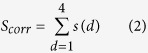


Thirdly, and last improvement, the solvent accessibility (SA) structural feature was appended in a SA profile. The SA of a protein residue is the surface area of a protein residue that is accessible to solvent. Solvent accessibility is a fundamental structural feature since it is related to the hydrophobic properties of residues. Hydrophobic force plays an important role during the folding process, affecting the protein packing and consequently the protein spatial arrangement^31^. Therefore, homologs sharing the same fold should also have similar SA patterns^27^,^32^.

The SA profile of the template is computed by discretizing the real value of relative solvent accessibility estimated by NACCESS in ten classes. The SA profile of the target is composed of the probabilities of the 10 solvent accessibility classes (from buried to exposed classes) predicted using the PROF software^27^ from the MSA at each position.

## Results and Discussion

### Assessments of ORION

**Figure 1:**
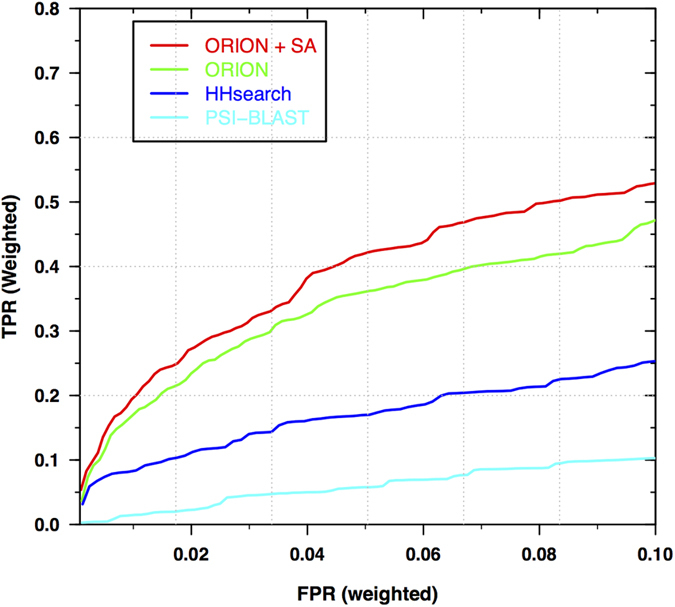
Performance of ORION, with original ORION approach (green^22^), ORION with solvent accessibility (SA, in red), HHsearch (in blue) and PSI-BLAST (in light blue) at detecting related proteins within the same fold levels for all pairs of the HOMSTRAD dataset. The false positive rate (FPR) and the true positive rate (TPR) are weighted to prevent compositional biases from dominating the benchmarks. For this purpose, each template and query is weighted with the number of members belonging to the same fold level.

This new version of ORION has been assessed on a benchmark including a balanced test set derived from the HOMSTRAD database containing 1032 targets. These improvements increase the true positive rate (TPR) of template detection by 5% compared to the initial version of ORION for 10% of false positive rate (FPR) (see Fig. 1). Indeed, at 10% of FPR, ‘ORION+SA’ reaches ~52% of TPR against ~47% of TPR for ORION without SA.

### ORION web server

#### Input and parameters

**Figure 2:**
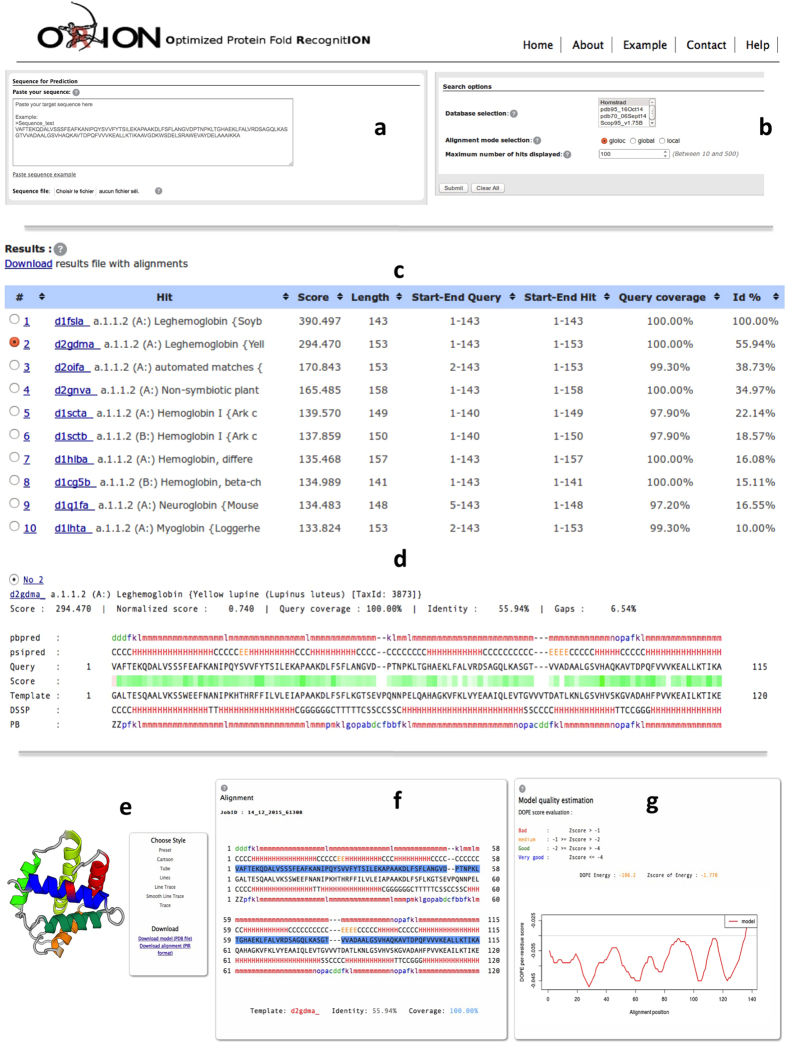
Overview of ORION web server. (**a**) Sequence/file submission in FASTA or plain text format. (**b**) Search options: Template database selection, alignment mode selection and number of hits to display. (**c**) Example of output results for the Leghemoglobin A protein (UniprotKB: P02238). (d) Query-template alignment results. (**e**) Model viewer. (**f**) Model-template alignment. (**g**) Global and local model quality estimation.

The user provides a protein query sequence in FASTA or plain text format (see Fig. 2a). The ORION web server accepts sequences between 15 and 1000 residues, but performs better on sequences containing no more than one protein domain. Therefore, multiple protein domains sequences should be ideally split into single protein domain. If the domain parts are not identified yet, user can use dedicated web servers for this purpose, like DOMAC^33^ or SEG-HCA^34^. Then, the user chooses the template databank, the alignment mode and the maximum number of hits to display. User can provide an e-mail to get the link to the results page (see Fig. 2b), which is optional but highly recommended since the process takes tenths of minutes if the queue is free but it can takes hours otherwise.

Three alignment modes are supported (‘gloloc’, ‘local’ and ‘global’). In ‘gloloc’ mode, the query profile is locally aligned along the entire length of the template profile. In ‘local’ mode, no penalties are added for begin/end gaps on both of the query and template profile and both can be locally aligned. In ‘global’ mode, query and template profile are entirely aligned. ORION is optimized for the ‘gloloc’ mode, since databank such as HOMSTRAD contain only protein domains and the query can have one or several domains. The ‘local’ mode is most suitable for a sensitive search with a large protein query sequence.

Users have the choice between five templates profiles databases obtained from three well-known databases: PDB^1^, SCOP^35^ and HOMSTRAD^36^ database (see Table 1). The PDB template database is based on the protein data bank, which contains all available 3D structures of proteins. SCOP template database is constructed from the manual classification of protein domains based on similarities of their structure and amino acid sequences. For the PDB and SCOP databases, sequence alignments were obtained by three iterations of PSI-BLAST on the non-redundant databank Uniref90^25^ with an E-value threshold of 10^−3^ and structure profiles were directly computed from the 3D coordinates of the protein chain/domain structure. Contrary to the PDB and SCOP databases, the HOMSTRAD template profiles database is based on structural alignments of homologous proteins. Since the structures of homologous proteins are generally better conserved than their sequences^19^, the HOMSTRAD template database should be most sensitive for detection of low homology relationships.

Once the input sequence has been entered and parameters selected, the user launches the job by clicking on the ‘submit’ button. The user is redirected to a waiting page, on which information of the status of the job is displayed and updated automatically every 30 sec. Contrary to other similar servers, ORION web server also includes an accurate prediction system of the waiting and queuing time. At the end, results are displayed on the same page.

### Results display

ORION results are displayed in a table of eight sortable columns containing template information matched by ORION such as the template description, the score, the corresponding template length, starting and ending residue numbers of the aligned query/template, the query coverage and the percentage of identity (see Fig. 2c). By default, templates are ranked using the ORION score but can be sorted according to other columns. Each template is linked to the PDB summary page that provides a description of the selected one.

The query-template alignments are displayed with the predicted/assigned PB elements and called “pbpred” for the predicted PBs of query sequence and “PB” for the assigned PBs of the template structure. Query and template secondary structure information that is predicted by PSIPRED software^37^ (‘psipred’) and assigned by DSSP software^38^ (‘DSSP’), are also shown for indicative purposes (see Fig. 2d). Secondary structure elements are colored in red and green for the two main types: α-helix and β-strand, respectively. PB elements are similarly colored, red for α-helix elements (central α-helix: *m* and α-helix N/C cap transitions: *f*, *k*, *l*, *n*, *o* and *p*) and in green for β-strand elements (central β-sheet: *d* and β-sheet N/C cap transitions: *b*, *c* and *e*). Finally, turn/coil elements are colored in blue (PBs *a*, *g*, *h*, *i* and *j*). PBs give an accurate description of the 3D structure using 16 local conformations, contrary to the secondary structure elements, which are composed of only 3 predicted states (α-helix, β-strand and coil). Therefore, PB helps user to analyze more precisely the local structure conformation of the query protein. User can also identify high scoring regions with the scores color scale, which correspond to the ORION scores between the compared positions^22^.

Additionally, user can select a template and build a protein model. ORION webserver displays the model obtained with MODELLER^39^ using the selected ORION query-template alignment. The 3D model can be explored thanks to the PV viewer JavaScript module^40^ and can be rendered with different styles (cartoons, tube, line, trace, see Fig. 2e).

The model-template alignment is shown with secondary structure and PB elements annotations. Hence, the user can link the regions of interest in the model and its local conformation (*e*.*g*. a gapped region corresponding to a coil-helix transition, see Fig. 2f). Finally, user can easily analyze the global and local quality of the model. For this purpose, global and local quality model estimation measures are shown using a graphical representation and an intuitive color scale (see Fig. 2g). The global model quality estimation is performed using the DOPE score calculation^41^ computed from all alpha carbons of the model. A global score of the model quality (z-score) is computed from the score of 50 decoys, which are obtained from random permutations of the amino-acid positions of the initial model. This score indicates the general compatibility of the model fold and its amino acid sequence. Scores greater than -1 are likely to be poor models. Scores between -1 and -2 indicate medium quality models, while scores between -2 and -4 are likely to be ‘reliable’ models. A score lower than -4 indicates a native-like model. For local measure, the DOPE score per residue, obtained from MODELLER, is plotted for each position of the alignment. This score is the mean value of the normalized DOPE score per residue over a sliding window of 15 residues. A gray line indicates the pseudo-energy threshold of 0, below which quality is considered as poor.

### Example

Since ORION uses accurate sequence/structural profiles, it is perfectly appropriate for remote protein homology detection. As an example, the sequence of T0818-D1 target from the eleventh Critical Assessment of Structure Prediction (CASP11) experiment^42^ was predicted. This 134 residues target corresponds to an NTF2-like (Nuclear Transport Factor 2-like) protein from *Eubacterieum siraeum* (PDB code: 4r1k). T0818-D1 belongs to the ‘hard target’ level in the ‘Template based modeling’ category. For this target, a preliminary version of ORION server named ‘Alpha-Gelly-Server’, ranked second among 44 servers. Here, we show an example of the structure prediction from this target sequence.

#### Identification of related proteins

The submitted job to ORION web server was done with the following parameters: the search is performed in the PDB95 database with the ‘gloloc’ alignment mode and a maximum of 100 hits in the results.

A summary hit list is displayed with the identified templates. All of these templates share a very low sequence identity with T0818-D1 (mean value is 8.45%; the maximum value equals to 14.63%). Nonetheless, some of the best ranked templates belong to the NTF2-like superfamily and so provides insights to the topology of T0818-D1. Protein sequences of NTF2-like superfamily are very diverse^43^ and thus are hard to detect based only on a simple sequence or sequence profile search. ORION has the advantage to use accurate structural features in profiles that allow identifying very remote homologous proteins. ORION succeeded to identify several NTF2-like proteins with very close scores. In the first 5 identified templates, we have selected the fourth template, which is the only template with 100% of the query coverage. This template corresponds to the crystal structure of the Putative scyalone dehydratase from *Novosphingobium aromaticivorans* (PDB code: 3ef8, chain A).

The T0818-D1-3ef8_A alignment shows a good agreement between predicted structural elements (‘psipred’ and ‘pbpred’, respectively) with those assigned from the template structure (‘DSSP’ and ‘PB’, respectively). Only a short region (from ~60 to ~75 positions) is problematic as it is predicted as a α-helix/coil while it is assigned as a β-strand in the template structure. The 3ef8_A template seems to be a suitable template for the homology modeling of T0818-D1 target.

#### 3D structure prediction

**Figure 3:**
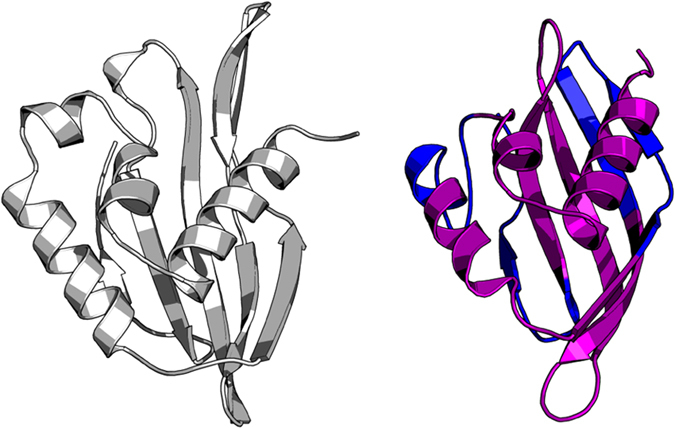
Example of the prediction of T0818-D1 structure with ORION webserver. Target and model structure of T0818-D1 are colored in gray and purple, respectively. The structures were aligned with the TM-align program^61^. The RMSD value between the two aligned structures is 3.89 Å. The low-quality zones are reported in blue in the model.

We create a 3D protein model using MODELLER with the T0818-D1-3ef8_A alignment, by clicking on the ‘Build 3D model’ button. The model obtained is composed of α-and β-regions organized in three α-helices followed by an antiparallel β-sheet of 5 β-strands (Fig. 3).

**Figure 4:**
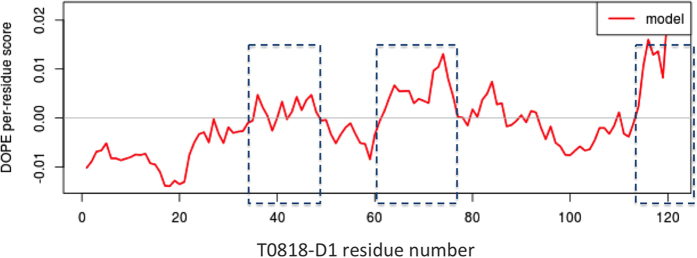
Normalized DOPE score per residue of the T0818-D1 model. A gray line indicates the zero value threshold above which, scores are likely to be poor. The normalized DOPE score is obtained with MODELLER and corresponds to the DOPE energy normalized over the number of DOPE restraints acting on each residue. Poor quality regions are delineated by blue squares and go from residue 35 to 47, 60–77 and from 115 to 132.

**Figure 5:**
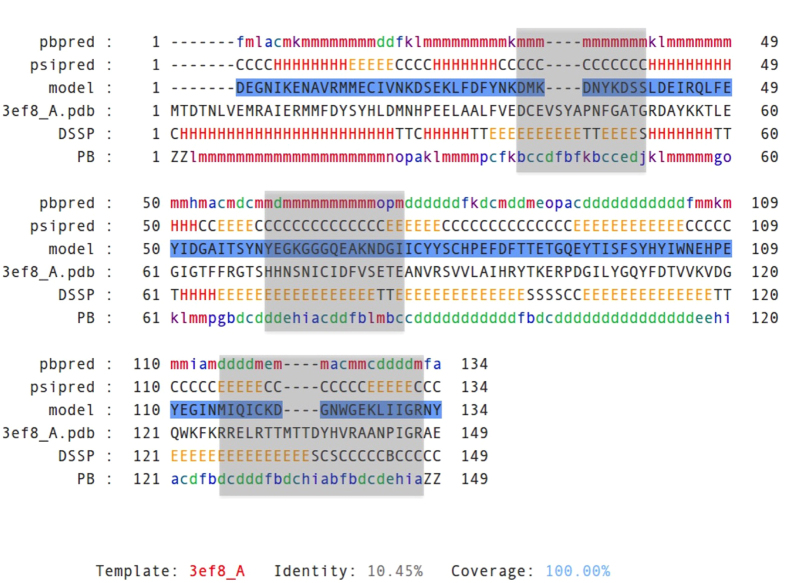
ORION query (T0818-D1) - template (3ef8_A) alignment with PB and secondary structures (SS) annotation. Predicted PB (“pbpred”) and SS (“psipred”) annotation is reported on the query/model sequence as “pbpred” and “psipred”, respectively. Assigned PB and SS annotation is reported on the template sequence as “PB” and “DSSP”, respectively. The sequence of the T0818-D1 model is colored in blue while the sequence of 3ef8_A is shown in black. Regions of high structural complexity in the template 3ef8_A that are in the vicinity of poor quality regions in the model are delineated by gray filled squares and located around residue 33, 67 and residue 122.

The overall quality of the model is estimated as ‘medium’ with a z-score between −1 and −2 and have a root mean square deviation (RMSD) value of 3.8 Å with the target structure. Thus, we investigate for the quality of local regions in the model. We notice 3 main low quality regions from residues 35 to 47; 60–77 and 115–132, in which the DOPE score per residue is over the threshold of 0 (Fig. 4, blue squares; Fig. 3, blue regions). The analysis of the template PB elements reveals that theses regions correspond to 3 β-strand regions of high complexity. Indeed, they are assigned as a succession of central beta elements (PB *d*) alternating with beta-coil transitions elements (PBs *b*, *c* and *e*) (Fig. 5, gray squares). This could not be revealed by the analysis of the secondary structure elements alone and highlights the importance of using PB instead of secondary structures. User can download the model as a PDB file and perform complementary analyses.

### Comparisons with other web servers

**Figure 6:**
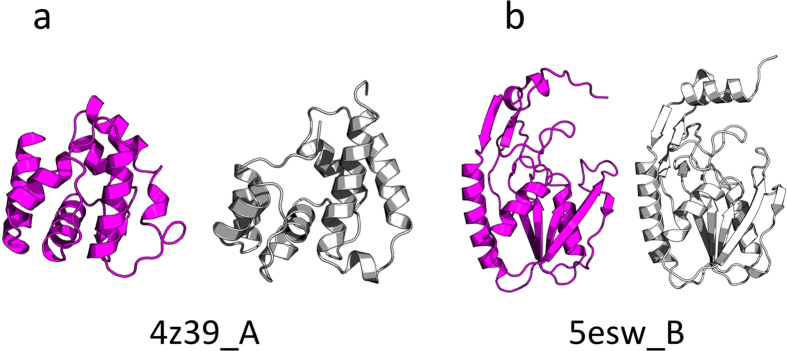
Prediction of 4z39_A (**a**) and 5esw_B (**b**) structures with ORION webserver. Models and targets structures are colored in purple and gray, respectively. The structures were aligned with the TM-align program^61^. The RMSD values between the targets and model structures are 5.48 Å and 3.37 Å, respectively.

**Figure 7:**
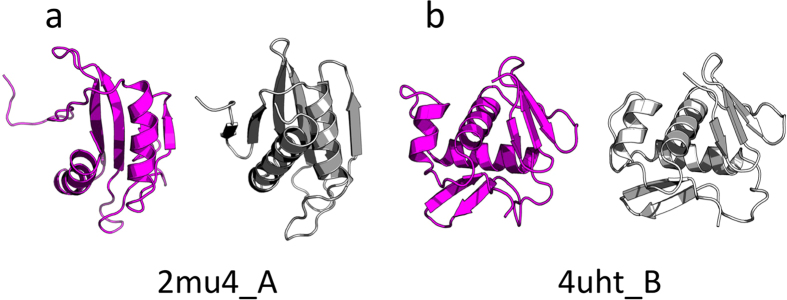
Prediction of 2mu4_A (**a**) and 4uht_B (**b**) structures with ORION webserver. Models and targets structures are colored in purple and gray, respectively. The structures were aligned with the TM-align program^61^. The RMSD values between the targets and model structures are 5.03 Å and 3.05 Å, respectively.

We show 4 examples from the Continuous Automated Model EvaluatiOn^44^ (CAMEO) server which provides a continuous evaluation of the accuracy and the reliability of protein structure prediction servers (Figs 6 and 7). For the 4 examples, ORION server results are compared to the results of the 11 web servers that are continuously assessed in CAMEO (Tables 2 and 3). The server list is composed of 4 single-method fold recognition techniques: the HHpred^45^, SPARKS-X^46^, RaptorX^47^, Princeton_TEMPLATE and Phyre2^48^ servers, two consensus-based fold recognition methods: the IntFOLD2-TS^49^ and IntFOLD3-TS^50^ servers, two *ab initio* and *de novo* approaches combined with fold recognition methods: the Robetta^51^ and RBO Aleph^52^ servers and two sequence search methods: the SWISS-MODEL^53^ and BLAST^7^ servers.

ORION models were generated using the first ranked template and we checked that the selected template has been released into the PDB before the CAMEO target date prediction, in order to compute models under the same conditions as during the target release date. Since the HHpred server^45^ and the SPARKS-X server^46^ have been assessed by CAMEO for two and three of the four examples, respectively, we have launched a prediction on HHpred and SPARKS-X server for the missing targets. For the HHpred server, the two missing models were obtained using the ‘pdb70_13Apr16’ template database with the default parameters and the ‘automatic template selection’ option. For the SPARKS-X server, the missing model was obtained with the default parameters and using the first ranked template. We also ensured that the HHpred and SPARKS-X models were based on templates that have been released into the PDB before the CAMEO target date.

The first example is an odorant binding protein (OBP3) from *Megoura viciae* (PDB code: 4z39, chain A), an all-α protein of 121 residues length, which is classified by CAMEO as ‘hard target’ (Fig. 6a). The best model was proposed by Robetta server^51^ with a TM-score^54^ of 0.66 and ORION model ranked second with a TM-score of 0.64. However, the ORION model was obtained after 22 minutes of computation contrary to Robetta server, which took 20 hours to predict the model (Table 2, left). The second example is a hydrolase (Apo hypoxanthine-guanine phosphoribosyltransferase) protein from *Legionella pneumophila* (PDB code: 5esw, chain B). 5esw_B is an α + β protein of 197 residues length that is classified as a medium target (Fig. 6b). The ORION server outperforms all the compared servers according to the ORION model that has the higher TM-Score (0.88). Since the SWISS-MODEL^53^ server has predicted an incomplete model with 89% of coverage, the ORION model has also the lowest RMSD value for the complete model (3.37 Å) (Table 2, right). The two other examples are of a medium level. The first is an α + β protein of 119 residues length from *Francisella tularensis* (PDB code: 2mu4, chain A) (Fig. 7a) and the second is a DNA binding domain of CpxR from *Escherichia coli* (PDB code: 4uht, chain B) of 102 residues length (Fig. 7b). According to the TM-score, ORION server has predicted the second best model of 2mu4_A (0.64) in only 21 minutes (Table 3, left). However, the ORION server does not perform as well as the other targets for 4uht_B. Indeed, the ORION model is ranked sixth over the 12 servers with a TM-Score of 0.81. The RBO Aleph^52^ model has the highest TM-score value (0.87) and the Robetta model, which is ranked second, has the lowest RMSD value (2.18 Å) (Table 3, right).

Based on these four examples, ORION server outperforms similar fold recognition servers based on different algorithms such as HHpred, SPARKS-X, RaptorX, Princeton_TEMPLATE and Phyre2. Robetta server is, with I-TASSER^55^ server, one of the most powerful and accurate tool for protein structure prediction^4^,^56^,^57^,^58^,^59^. However, these servers are based on *ab initio* and *de novo* methods, which are more time-consuming.

## Conclusion

The ORION server is a tool for homology detection and template-based modeling. Based on hybrid profiles combining sequence and structural information, ORION web server is very sensitive and able to detect remote homologous proteins that cannot be reached by other tools such as BLAST^60^, PSI-BLAST^7^ or HHsearch^16^. Comparisons with similar servers show that ORION web server is also a powerful tool for the protein structure prediction. However, since the PB prediction system has been optimized for globular proteins, the performances of ORION for transmembrane proteins are not as reliable as for globular proteins. Thus, further improvements would be possible by developing a PB prediction system dedicated to transmembrane proteins. This server offers a user-friendly interface combining a fast and sensitive approach. The web server generally takes a few dozen minutes to return a prediction.

## Additional Information

**How to cite this article**: Ghouzam, Y. *et al*. ORION: a web server for protein fold recognition and structure prediction using evolutionary hybrid profiles. *Sci. Rep.* **6**, 28268; doi: 10.1038/srep28268 (2016).

## Figures and Tables

**Table 1:** List and description of the databases used in the ORION webserver.

Database	Ref	Description
PDB95 or PDB70	30	A collection of ORION templates profiles based on the protein data bank (PDB), which contains all available 3D structures of proteins, filtered with a maximum sequence identity of 95% or 70%.
scope95 or scope70	31	A collection of ORION templates profiles of SCOPe domains sequences/structures. A filtered version of the SCOPe sequences set to 95%/70% maximum sequence identity from ASTRAL website.
HOMSTRAD	32	A collection of ORION templates profiles obtained from HOMSTRAD families (aligned sequences and structures) from the HOMSTRAD website.

**Table 2:** Structure prediction results of 4z39_A (left) and 5esw_B (right) targets.

Method	Resp. time	Cov %	Rmsd Å	TM-Score	Method	Resp. time	Cov %	Rmsd Å	TM-Score
Robetta	20:02:31	100	5.23	0.66	ORION	00:31:52	100	3.37	0.88
ORION	00:22:02	100	5.48	0.64	Robetta	16:36:12	95	3.57	0.87
SPARKS-X	01:44:23	100	4.39	0.62	RaptorX	12:29:01	100	3.68	0.86
IntFOLD2-TS	00:39:38	100	5.68	0.61	Princeton_TEMPLATE	01:03:10	100	3.62	0.85
IntFOLD3-TS	17:11:36	100	5.98	0.61	RBO Aleph	04:22:19	100	5.93	0.85
Phyre2	01:17:32	95	4.17	0.60	SPARKS-X	01:21:07	100	5.31	0.85
RaptorX	03:23:19	100	5.65	0.60	IntFOLD3-TS	04:46:19	100	4.81	0.84
HHpred*	00:03:27	100	7.73	0.59	SWISS-MODEL	00:14:13	89	2.12	0.84
Princeton_TEMPLATE	03:38:25	100	5.75	0.59	IntFOLD2-TS	05:49:21	100	5.19	0.83
RBO Aleph	02:08:36	100	6.18	0.58	HHpred*	00:02:11	100	5.19	0,82
SWISS-MODEL	00:02:32	88	6.41	0.45	Phyre2	00:41:13	93	4.73	0.81
NaiveBLAST	00:00:18	32	2.67	0.25	NaiveBLAST	00:00:01	83	5.69	0.73

ORION webserver predictions results were compared to 11 servers in CAMEO. Results of the 11 servers were taken from the CAMEO server. The table describes the response time (Resp. time) in hours:minutes:seconds, the percentage of coverage of the model and target (cov), the RMSD value between the model and the target (in Å) and the TM-score for the 12 servers compared. The ORION server is in bold and stars ‘*’ indicate that the model is obtained manually from the considered webserver.

**Table 3:** Structure prediction results of 2mu4_A (left) and 4uht_B (right) targets.

Method	Resp. Time	Cov %	Rmsd Å	TM-Score	Method	Resp. Times	Cov %	Rmsd Å	TM-Score
Robetta	22:17:39	94	5.66	0.61	RBO Aleph	02:39:54	100	3.13	0.87
ORION	00:21:32	97	5.03	0.55	Robetta	06:00:50	100	2.18	0.85
SPARKS-X*	00:23:21	100	6.18	0.55	RaptorX	12:18:38	100	3.02	0.84
RaptorX	22:42:28	100	6.29	0.51	SPARKS-X	00:26:43	100	3.24	0.82
Princeton_TEMPLATE	02:54:36	100	8.41	0.50	HHpred*	00:08:12	100	3.15	0.82
RBO Aleph	00:05:45	100	15.55	0.44	ORION	00:24:01	100	3.05	0.81
IntFOLD2-TS	19:31:46	100	13.35	0.31	IntFOLD3-TS	17:13:54	100	3.28	0.81
HhpredB	00:02:39	100	12.97	0.30	IntFOLD2-TS	13:51:57	100	3.21	0.80
IntFOLD3-TS	01:40:45	100	21.90	0.22	Princeton_TEMPLATE	03:00:45	100	3.10	0.80
NaiveBLAST	00:00:28	60	15.86	0.19	SWISS-MODEL	00:02:51	96	3.22	0.80
Phyre2	00:09:40	15	3.66	0.13	NaiveBLAST	03:22:18	96	2.78	0.78
SWISS-MODEL	00:01:40	63	14.85	0.13	Phyre2	00:28:51	97	5.17	0.71

ORION webserver predictions results were compared to 11 servers in CAMEO. Results of the 11 servers were taken from the CAMEO server. The table describes the response time (Resp. time) in hours:minutes:seconds, the percentage of coverage of the model and target (cov), the RMSD value between the model and the target in Ångströms and the TM-score for the 12 servers compared. The ORION server is in bold and stars ‘*’ indicate that the model is obtained manually from the considered webserver.
